# Cytotoxic Effects of Some *Nepeta* Species against Breast Cancer Cell Lines and Their Associated Phytochemical Properties

**DOI:** 10.3390/plants11111427

**Published:** 2022-05-27

**Authors:** Esra Köngül Şafak, Gökçe Şeker Karatoprak, Tuncay Dirmenci, Hayri Duman, Nurgün Küçükboyacı

**Affiliations:** 1 Department of Pharmacognosy, Faculty of Pharmacy, Erciyes University, Kayseri 38280, Turkey; gskaratoprak@erciyes.edu.tr; 2 Department of Biology Education, Necatibey Education Faculty, Balikesir University, Balikesir 10010, Turkey; dirmenci@balikesir.edu.tr; 3 Department of Biology, Faculty of Science, Gazi University, Ankara 06560, Turkey; hduman@gazi.edu.tr; 4 Department of Pharmacognosy, Faculty of Pharmacy, Cyprus University of Health and Social Sciences, Güzelyurt, TRNC, Mersin 33190, Turkey; nurgun.kucukboyaci@kstu.edu.tr

**Keywords:** *Nepeta*, antiproliferative effect, ursolic acid, triterpenoid

## Abstract

*Nepeta* is one of the largest genera of the Lamiaceae family. *Nepeta* species are commonly employed in traditional medicine for a variety of ailments, as well as food additives. In addition, they also come to the fore with their rich phytochemical content. In the present study, the quantitative phytochemical content of methanolic extracts and infusions prepared from the aerial parts of 14 *Nepeta* taxa collected from Turkey and their cytotoxic effects on two breast cancer cell lines, MCF-7 and MDA-MB-231, were investigated by using the MTT (3-(4,5-dimethylthiazol-2-yl))-2,5-diphenyltetrazolium-bromide) test. According to HPLC-PDA analysis, *N. racemosa* methanolic extract had the highest ursolic acid content with 165.9 mg/g extract. Total sterol, total iridoid, and total triterpenoid content were determined to be greatest in the methanolic extracts of *N. meyeri, N. trichocalyx* and *N. phyllochlamys*. The MTT experiment demonstrated that certain *Nepeta* species suppressed the growth of MCF-7 and MDA-MB-231 cells in a dose-dependent manner. Statistical analysis revealed a strong correlation between the cytotoxic effects of the extracts and their triterpene content. In conclusion, the data obtained from this study are important in terms of forming a basis for advanced anticancer activity studies on breast cancer with *Nepeta* sp.

## 1. Introduction

*Nepeta* L. is one of the largest genera of the Lamiaceae family, which is represented by approximately 300 species worldwide and is generally distributed in Central and Southern Europe, the Near East, Central and South Asia, and some parts of Africa [1]. It is represented in Turkey by 44 taxa, 20 of which are endemic [2]. Species belonging to the genus have different uses such as traditional medicine, agrochemical, and food additives [3]. It has been reported that the fresh or dried flowering top leaves of *Nepeta cataria* are used as flavoring agents in dishes, especially sauces, soups, and cheeses [3,4]. Because of their antispasmodic, expectorant, diuretic, antiseptic, emenagogue, antitussive, antiasthmatic, anticonvulsant, sedative, diaphoretic, analgesic, antipyretic, antifungal, antiviral, anti-inflammatory, antihemorrhoidal, antirheumatic activities *Nepeta* species are commonly used to treat a variety of ailments in the regions where they grow [5,6,7,8]. Numerous phytochemical studies with the genus have shown that the main constituents of *Nepeta* species are nepetalactones, iridoids, and their glycosides as well as diterpenes, triterpenes, and flavonoids. Moreover, most *Nepeta* species are rich in essential oils [9,10,11]. In parallel with their rich chemical content, including medicinally important compounds, and traditional uses, plants belonging to the genus have been shown to have many important biological activities. In respect to several in vitro and in vivo studies, different *Nepeta* species have been reported to possess antitumoral [12], anticancer [13], antiangiogenic [14], cytotoxic [15], antibacterial [16], antiparasitic [17], antioxidant [18], anti-Alzheimer [19], allelopathic [20], immunomodulator [21], antiaggregant [22], insecticide [23], spasmolytic, bronchodilator [24], anxiolytic [25] and antidiabetic [26] activities.

Breast cancer is the second most common type of cancer in the world after lung cancer and is a common malignancy in women. Breast cancers can be classified according to stage, pathology, grade, and expression of estrogen receptor (ER), progesterone receptor (PR), or human epidermal growth factor receptor (Her2/neu) [27]. Two types of breast cancer cells that have attracted attention by researchers for their differences are MDA-MB-231 and MCF-7. Both MCF-7 and MDA-MB-231 cells, despite being invasive ductal/breast carcinoma cells, represent a conspicuous example as they have phenotypic/genotypic differences: while MCF-7 is hormone-dependent (negative for HER-2 receptor, positive for estrogen and progesterone receptor), MDA-MB-231 is triple negative [28].

The traditional use of *Nepeta betonicifolia* against cancers in the form of a decoction or powder was documented in an earlier ethnobotanical study carried out in Turkey [29]. In addition, in a study conducted with *Nepeta binaloudensis*, it was reported that the extracts prepared from its aerial part decreased the cell viability on breast cancer cell lines (MCF-7 and MBA-MD-231) in a dose- and time-dependent manner [30]. These studies have encouraged us to investigate the effects of 14 *Nepeta* taxa on MCF-7 and MDA-MB-231 cell lines. These species included: *Nepeta argolica* Bory & Chaub. subsp. *tumeniana* (Dirmenci), *Nepeta trichocalyx* Greuter & Burdet, *Nepeta cadmea* Boiss., *Nepeta crinata* Montbret et Aucher ex Benth., *Nepeta humilis* Benth., *Nepeta italica* L., *Nepeta leptentha* Boiss. et Hauskn., *Nepeta meyeri* Benth., *Nepeta phyllochlamys* P. H. Davis, *Nepeta recemosa* Lam., *Nepeta stricta* (Banks & Sol.) Hedge et Lamona var. *curvidens* (Boiss. et Bal.) Hedge et Lamond, *Nepeta stricta* (Banks & Sol.) Hedge et Lamona var. *stricta* (Banks et Sol.) Hedge et Lamond, *Nepeta teucriifolia* Willd., and *Nepeta transcaucasica* Grossh.

The presence of diverse classes of terpenes in different species of the genus *Nepeta* and some data related to the cytotoxicity of some of these compounds such as nepetalactone (iridoid), ursolic acid (triterpene), and nepetolide (diterpene) directed us to examine total sterol, total iroid, and total triterpenoid content of these species [31,32,33]. In addition, ursolic acid content was quantitatively analyzed by HPLC-PDA. Finally, Spearman’s correlation analysis was performed in order to correlate the observed biological effects with the phytochemical content of the species. The biological activities and contents of the majority of the species we investigated are not found in the literature and were evaluated in depth in this study for the first time. The scientific information acquired from this study is likely to contribute to the current database on the biochemical characteristics of *Nepeta* species.

## 2. Results and Discussion

### 2.1. Phytochemical Analysis

*Nepeta* species attract attention with their rich phytochemical content, especially iridoid glucosides (ajugol, aucubin, cyclopentane-monoterpene 1,5,9-epideoxyloganic acid, ixoroside, monoterpene enol acetates, nepetariaside, nepetanudosides, nepetaside, velpetin), iridoid monoterpenes (nepetalactones, dihydronepetalactones, iridomyrmecin, isoiridomyrmecin, nepetalic acid), monoterpenes (1,8-cineole, linalool, nerol, geranial, etc.), sesquiterpenes (*β*-caryophyllene, germacrene D, *β*-farnesene, α-humulene, nehipetol, nehipediol, etc.), diterpenes (netidiol A, teideadiol, isopimarol, parnapimarol, nepetaparnone, crassifol, etc.), triterpenes (ursolic acid, oleanolic acid, lupeol, *β*-amyrin, triterpenes nepetalic acids, nepeticin, betulin, nepehinol, etc.), flavonoids (cirsimaritin, isothymusin, genkwanin, luteolin, apigenin, salvigenin, etc.), phenolic acids (rosmarinic acid, caffeic acid, ferulic acid, chlorogenic acid, coniferin, p-coumaric acid, gallic acid, vanillic acid, etc.), and steroid derivative (*β*-sitosterol, stigmasterol etc.) compounds [34,35,36].

There are various phytochemical studies in the literature regarding some of the species we included in our study.

It has been reported that *Nepeta cadmea*, one of the species we are concerned with, contains benzoic acid derivatives such as rosmarinic acid, caffeic acid, chlorogenic acid, ferulic acid, flavonoids such as apigenin and rutin, iridoid monoterpenes, iridoid, and eugenol glycosides [34,35,36,37].

Similarly, an investigation about analysis of *N. humilis* extracts with RP-HPLC-DAD showed that the major component in all parts of the plant, especially in flowers, is rosmarinic acid, while flowers contain phenolic compounds such as chlorogenic acid, luteolin and apigenin [38].

In *N. transcaucasica*, flavonoid derivative compounds such as apigenin, cirsimaritin, cosmosiin, gardenin B, genkwanin, hispidulin 7-glucoside, nepetrin, (Luteolin 7-*O-β*-D-glucopyranoside), Luteolin 7-*O-β*-D-glucuronide, salvigenin, scutellarin A (apigenin 7-*O-β*-D-glucuronide), xanthomicrol, nepetin 7-glucuronide, and hispidulin 7-*O-β*-D-glucuronide were previously identified [34].

Some articles on *N. italica* have shown that this plant contains various monoterpenes, stigmasterol, ergosterol, and betasitosterol, as well as phenolic compounds (morin, catechin, naringin) [35,39].

In various studies, spathulenol (sesquiterpene), and teucriifolian A-C (iridoite) were identified in *N. teucriifolia*, nepetalactone derived iridoid monoterpenes in *N. meyeri*, and iridoid glycosides named nepetaracemosides A and B in *N. racemosa* besides nepetalactones [35,40,41,42].

Various monoterpenes have been also detected in *N. lepthenta* and *N. phyllochlamys* species with some studies, but a detailed phytochemical investigation has not been found in the literature, except essential oil analyses of them. Similarly, no research has been found on the phytochemical properties of *N. aristata* and *N. crinita* species, except for their fatty acid content [35,43,44].

No research has been found based on the phytochemicals of *N. argolica* subsp. *tumeniana*, but there are some articles indicating the presence of argolic acid A, argolic methyl ester B, 1*α*, 2*β*, 5*α*-nepetonic acid, 1,5,9-epideoxyloganic acid, nepetalactones, monoterpenes, and sequiterpene derivatives in *N. argolica* subsp. *argolica* [35,45].

To our knowledge, neither *Nepeta stricta* var. *stricta* nor *Nepeta stricta* var. *curvidens* have been evaluated for their chemical composition or biological activity. The records in the literature about these species include a limited number of articles focused on their anatomical and morphological features.

Within the scope of the present study, total sterol, total iroid, and total triterpenoid assays of the above-mentioned species were performed and their ursolic acid content was also analyzed by HPLC-PDA. According to the phytochemical analyses, the total sterol content of *N. meyeri* methanolic extract was found to be the highest in the quantitative analysis, with 403.09 ± 15.24 mg cholesterol equivalents/g extract. Iridoid presence was detected in both methanolic extracts and infusions of *N. trichocalyx* and *N. crinita* species. The methanolic extract of *N. stricta* var. *curvidens*, on the other hand, contained iridoid, whereas the infusion did not. In contrast, iridoid appeared to be present in the infusion of *N. racemosa*, but not in the methanolic extract. Among these extracts, the total amount of iridoid was found to be highest in the *N. trichocalyx* methanolic extract with 85.60 ± 4.45 mg aucubin equivalents/g extract. The methanolic extract of *N. stricta* var. *curvidens* had the highest total amount of triterpenoids, with 928.07 ± 22.26 mg of oleanolic acid equivalents/g extract. Infusions of *N. transcaucasica* were found to be devoid of triterpenoids (Table 1).

**Figure 1:**
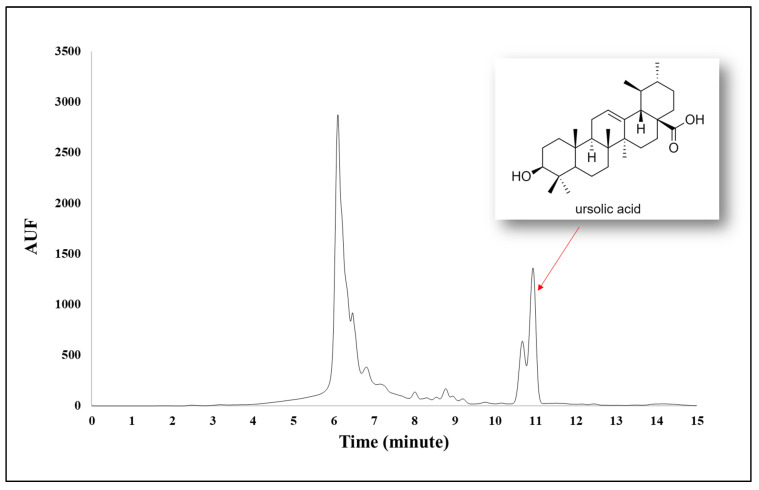
HPLC chromatogram of *N. racemosa* methanolic extract.

In addition, the amounts of pentacyclic triterpene ursolic acid in the extracts were investigated by HPLC-DAD analysis (Table 2). As a result of the analysis, while ursolic acid could not be detected in the infusions, it was shown that *N. racemosa* methanolic extract had the highest ursolic acid content with 165.9 ± 0.6 mg/g extract (Figure 1).

The presence of ursolic acid has been reported previously in many *Nepeta* species such as *N. binaludensis*, *N. aragonensis*, *N. cataria*, *N. clarkei*, *N. crassifolia*, *N. eriostachya*, *N. faassenii*, *N. grandiflora*, *N. heliotropifolia*, *N. juncea*, *N. leucophylla*, *N. mussinii*, *N. nuda* ssp. *albiflora*, *N. obtusicrena*, *N. pannonica*, *N. prattii*, and *N. teydea.* However the findings in the present study are the first examination (to our knowledge) on ursolic acid content of the species included in the study.

### 2.2. Cytotoxic Effect of Extracts

The MTT assay indicated that some *Nepeta* species inhibited the proliferation of MCF-7 and MDA-MB-231 cells in a dose-dependent manner. In MCF-7 cells, among the methanol extracts, *N. argolica*, *N. stricta* var. *curvidens*, *N.leptentha*, *N. phyllochlamys*, *N. racemosa*, and *N. transcaucasica* showed stronger cytotoxic activity than other extracts. All methanolic extracts were found to have significant antiproliferative activity compared to the control at the highest concentration (*p* < 0.001). *N. racemosa* and *N. stricta* var. *curvidens* showed activity with a significance of *p* < 0.01 at a concentration of 62.5 µg/mL. IC_50_ values of the active species were calculated as follows: *N. argolica* 186.66 ± 13.32 µg/mL, *N. stricta* var. *curvidens* 213.14 ± 17.65 µg/mL, *N. leptentha* 190.99 ± 13.41 µg/mL, *N. phyllochlamys* 79.91 ± 9.47 µg/mL, *N. racemosa* 61.38 ± 1.38 µg/mL, and *N. transcaucasica* 220.66 ± 9.03 µg/mL. It was determined that infusion extracts did not have as much activity as methanolic extracts in the same cell line. *N. racemosa* was the most potent infusion extract inhibiting viability by approximately 34% at 62.5 µg/mL concentration (*p* < 0.05). While cisplatin used as a positive control affected the viability with a significance of *p* < 0.001 in the range of 31.25–250 µg/mL, ursolic acid showed an antiproliferative effect with a significance of *p* < 0.01 even at 3.9 µg/mL. The methanolic extract of *N. racemosa*, the extract with the highest ursolic acid content, showed the highest cytotoxic effect in the MCF-7 cell line (Table 2).

*N. argolica*, *N. phyllochlamys*, *N. racemosa*, and *N. transcaucasica* showed more pronounced antiproliferative activity in the MDA-MB-231 cell line. IC_50_ values were 166.06 ± 7.9 µg/mL, 90.80 ± 1.67 µg/mL, 94.26 ± 2.85 µg/mL, and 178.07 ± 9.81 µg/mL, respectively (Table 3). It was observed that methanolic extracts of *N. phyllochlamys* and *N. racemosa* significantly decreased the viability in this cell line at a concentration of 125 µg/mL (*p* < 0.01). It was ascertained that infusion extracts in the same cell line did not have a significant viability-reducing effect. Infusion extracts showed antiproliferative effect at a concentration of 250 µg/mL with a significance of *p* < 0.05 were *N. cadmea*, *N. crinata*, *N. italica*, and *N. phyllochlamys*. In the MDA-MB-231 cell line, cisplatin was able to significantly inhibit viability at all concentrations studied (*p* < 0.001), while ursolic acid did not affect viability at concentrations of 0.975 µg/mL and 1.95 µg/mL compared to the control.

MCF-7 is an estrogen receptor positive (ER+) cancer cell line and is a good model for identifying the molecular events of some ER+ human breast cancers. The MDA-MB-231 (estrogen receptor-negative; ER−) cell line is more aggressive and does not respond to anti-estrogens. Chemopreventive compounds must have stronger toxicity in order to exhibit an antiproliferative effect in this cell line [46,47]. According to the study results, the IC_50_ values of the extracts except for *N. argolica* and *N. transcaucasica,* were found to be lower in the MCF-7 cell line. Ursolic acid likewise exhibited lower activity in the MDA-MB-231 cell line. Saponins and terpenoids in the composition of the extracts are thought to be responsible for the cytotoxic activity, and studies have shown that these compounds are cytotoxic [48,49,50].

As a consequence of the investigation into the literature, it was determined that the cytotoxic activities of the essential oils of *Nepeta* species were studied in different cancer cell lines [51,52,53,54]. Few studies have been conducted to assess the cytotoxic activities of *Nepeta* species extracts. It has been reported that *Nepeta deflersiana* chloroform extract does not affect viability in the MCF-7 cell line in the range of 10–250 μg/mL [13]. In the study by Orfali et al., it was stated that *N. deflersiana* ethyl acetate and *n*-butanol extracts were not cytotoxic against SK-OV-3, SK-MEL, KB, and BT-549 cancer cell lines [55]. In another study with the aerial parts of *Nepeta teucriifolia*, extracts were prepared with solvents of different polarities and their antiproliferative activities against C6 and HeLa cell lines were evaluated. Three new iriodite teucriifolian A-C were isolated from the active extract and their antiproliferative effects on HeLa and C6 cells were also evaluated. Among these compounds, only teucriifolian B compound at a dose of 250 μg/mL has been reported to have moderate antiproliferative activity against HeLa cells [40]. The toxicity profiles of ethanol, water, and *n*-hexane extracts of *Nepeta binaloudensis* in MCF-7, MBA-MD-231 cancer cells, and healthy MCF-10A cells were investigated. According to the findings, the most effective extract was *n*-hexane extract, with an IC_50_ value of 60.89 μg/mL for MCF-7, 92.18 μg/mL for MBA-MD-231, and >300 μg/mL for MCF-10A evaluated for 48 h. It was concluded that *N. binaloudensis* extract was more effective in MCF-7 cells, and this result was consistent with our results [30].

### 2.3. Correlations between the Phytochemical Content and Cytotoxic Activities of Extracts

**Figure 2:**
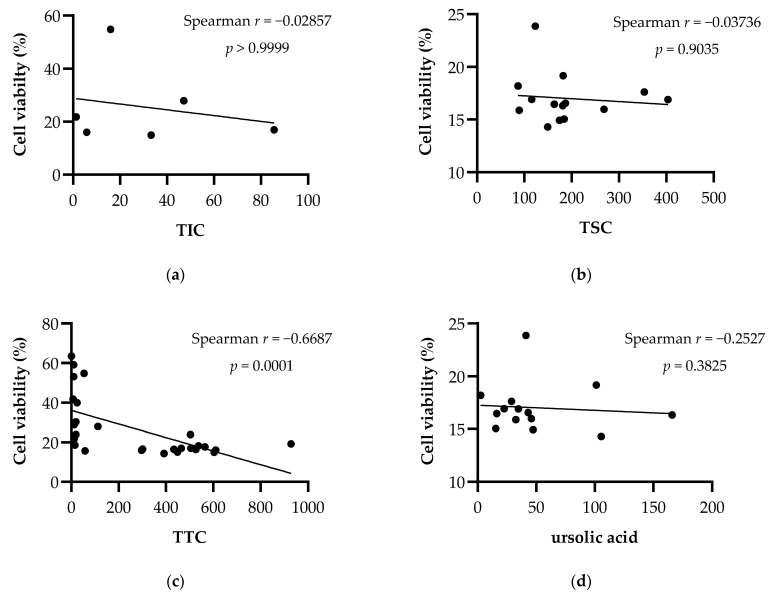
Spearman’s correlation between cytotoxic effects of extracts against MCF-7 cells and their phytochemical content. (**a**) TIC: Total iridoid content (mg aucubin equivalents/g extract). (**b**) TSC: Total sterol content (mg cholesterol equivalents/g extract). (**c**) TTC: Total triterpenoid content (mg oleanolic acid equivalents/g extract). (**d**) Ursolic acid (mg/g extract).

**Figure 3:**
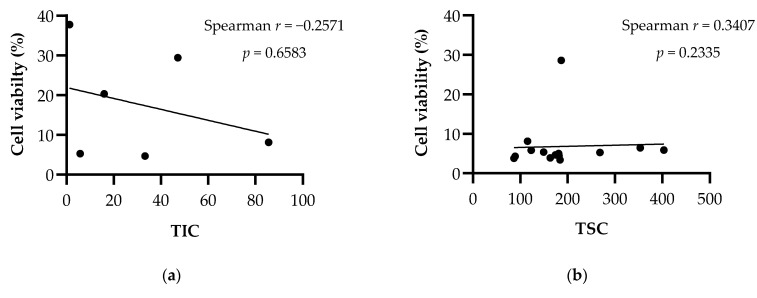

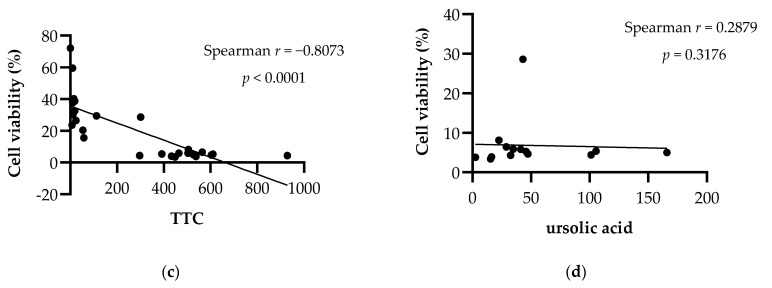
Spearman’s correlation between cytotoxic effects of extracts against MDA-MB-231 cells and their phytochemical content. (**a**) TIC: Total iridoid content (mg aucubin equivalents/g extract). (**b**) TSC: Total sterol content (mg cholesterol equivalents/g extract). (**c**) TTC: Total triterpenoid content (mg oleanolic acid equivalents/g extract). (**d**) Ursolic acid (mg/g extract).

The relationship between the phytochemical content of *Nepeta* extracts and their effects on cell viability in breast cancer cell lines was evaluated with the nonparametric Spearman’s correlation test and the results are shown in Figure 2 and Figure 3. According to the statistical evaluation, there is no significant correlation between the total iridoid, total sterol and ursolic acid amounts of the extracts, and their cytotoxic effects. Spearman’s ranking analysis revealed a strong correlation between the triterpenoid amounts of the extracts and their cytotoxic effects in both breast cancer cell lines MCF-7 (*r* = −0.6687; *p* = 0.0001) and MDA-MB-231(*r* = −0.8073; *p* < 0.0001).

Triterpenes are important natural compounds with anti-cancer and chemopreventive effects. They not only inhibit the survival of neoplastic cell lines, but also induce apoptosis in cancer cells, without affecting normal cells [56].

**Figure 4:**
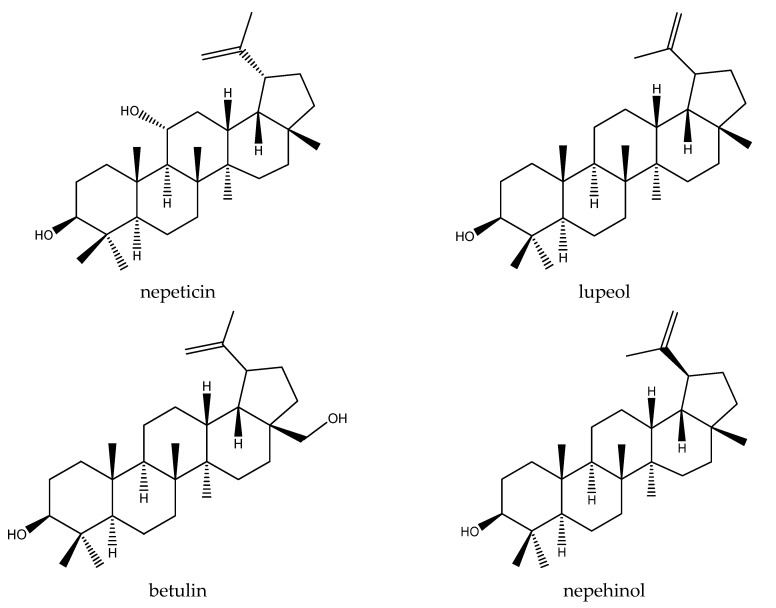
Chemical structure of some important triterpenes defined in genus *Nepeta*.

The most common triterpene identified in the genus *Nepeta* is ursolic acid. In addition, nepeticin, oleanolic acid, lupeol, betulin, nepehinol and triterpene nepetalic acids are among the important triterpenic compounds defined in this genus (Figure 4) [36]. No correlation was found between the cytotoxic effects of the extracts and their amounts of ursolic acid, but the methanolic extract of *N. racemosa*, which has the highest ursolic acid content among all samples, showed the highest cytotoxic effect in MCF-7 cells. *N. phyllochlamys* extract, which has the highest triterpene content, strongly inhibited cell proliferation in both cell lines. This shows that although ursolic acid contributes to the cytotoxic effect, it cannot be held responsible for the effect alone, and other compounds, especially other triterpenic structures in the extracts, also contribute to the effect.

## 3. Materials and Methods

### 3.1. Plants Materials and Preparation of Plant Extracts

In this study, 14 species of *Nepeta* were collected from different sites in Turkey and the identities of the species were confirmed by Dr. Tuncay Dirmenci and Dr. Hayri Duman. The powdered flowering aerial parts of the plants were weighed at 50 g and macerated with methanol (MeOH) (3 × 200 mL) at room temperature. After maceration, the methanolic extract was combined by filtration and was distilled in an evaporator. To prepare the infusion, 500 mL of boiling water was added to 10 g of the herb, boiled for 5 min and then filtered while hot. The extracts concentrated in the evaporator were dried with a lyophilizer. The prepared extracts were stored in the refrigerator at +4 °C.

In this report, plant names were abbreviated as NAT (*Nepeta argolica* subsp. *tumeniana*), NTX (*Nepeta trichocalyx*), NCD (*Nepeta cadmea*), NCR (*Nepeta crinata*), NHM (*Nepeta humilis*), NIT (*Nepeta italica*), NLP (*Nepeta leptentha*), NMY (*Nepeta meyeri*), NPH (*Nepeta phyllochlamys*), NRC (*Nepeta racemosa*), NSC (*Nepeta stricta* var. *curvidens*), NSS (*Nepeta stricta* var. *stricta*), NTC (*Nepeta teucriifolia*), and NTR (*Nepeta transcaucasica*).

### 3.2. Phytochemical Analysis

#### 3.2.1. Total Triterpenoid Assay

The total triterpenoid content was investigated using the method by Zhang et al., (2010). According to this method, vanillin-glacial acetic acid (5%, *w*/*v*, 0.5 mL) and 1 mL perchloric acid were added to the sample solution (0.5 or 2 mg/mL; 500 µL) and mixed in a test tube. It was left to cool in an ice bath for 15 min and then 5 mL of glacial acetic acid was added and mixed. After standing for 6 min, absorbances at 538 nm were measured. The total triterpenoid content of the extracts was calculated with a calibration curve obtained with oleanolic acid solution and the results were given as equivalents (OAE, mg/g extract) to oleanolic acid [57].

#### 3.2.2. Total Iridoid Assay

The total iridoid content was determined by the Trim–Hill test. According to this assay, 400 µL of the extract was treated with 4 mL of Trim–Hill reagent for 5 min at 100 °C. At the end of the reaction, absorbances were measured at 609 nm. Aucubin was used as standard. Total iridoid content of the extracts was calculated as mg aucubin equivalent/g extracts [58].

#### 3.2.3. Total Sterol Assay

The total sterol content was determined by Liebermann–Burchard assay. The extracts were stirred with chloroform and filtered. The Liebermann–Burchard reagent was prepared by adding 5 mL of concentrated H_2_SO_4_ to 50 mL of acetic anhydrous incubated in an ice bath for 30 min [59,60]. The quantitative assay was carried out according to the method reported by Johnson et al. (2020). Briefly, after mixing the sample solutions with Liebermann–Burchard reagent for 1 min, they were kept at room temperature (26 °C) for 13 min. Absorbances were measured at 650 nm and cholesterol was used as a standard. The total sterols content of the extracts was calculated as mg cholesterol equivalent/g extracts [61].

#### 3.2.4. Quantitation of Ursolic Acid with HPLC Analyses

The PDA detector combined with an Agilent 1100 liquid chromatography system was used to perform a qualitative and quantitative ursolic acid content analysis of the extracts. In the analysis, Thermo C18 analytical column (250 × 4.6 mm, 5 µm particle diameter) as the stationary phase, acetonitrile: methanol (80:20) system was preferred as the mobile phase. The mobile phase passed through the system isocritically at a flow rate of 1 mL/min and the total analysis time for one sample was set for 15 min. The detection wavelength was 210 nm. Solutions were prepared at concentrations ranging from 7.8–2000 µg/mL to obtain a calibration curve for ursolic acid (Sigma Chemical Company, St. Louis, MO, USA) purchased as standard. The regression equation was obtained as *y* = 8.9087*x* + 143.24 and the calibration plot showed good linearity with a correlation coefficient of 0.9998. Limits of detection (LOD) and limit of quantification (LOQ) of the ursolic acid standard were found 0.045 and 0.136 mg/mL respectively. Extracts were prepared at a concentration of 10 mg/mL, filtered, and then injected into the system. Each analysis was repeated three times [62].

### 3.3. Cell Culture

To investigate the cytotoxic effects of the extracts on breast cancer cells, two different human breast adenocarcinoma cell lines, MCF-7, and MDA-MB-231 were used. For both cell lines, DMEM containing 10% FBS (fetal bovine serum), and 1% penicillin-streptomycin were used as the medium. Cells cultured in the flask were passaged at the end of the two-day incubation period.

### 3.4. MTT Assay

The cytotoxic effect of the extracts on MCF-7, and MDA-MB-231 cells was determined by the colorimetric MTT method. After the cells in the flask were counted, they were distributed into 96-well microplates and then incubated in a CO_2_ incubator at +37 °C. At the end of 24 h, the supernatant of the cells was discarded and 100 µL of the sample was added to the microplate wells at concentrations varying between 3.906–1000 µg/mL for extract and 0.975–250 µg/mL for ursolic acid and cisplatin (as a positive control), and then exposed for 48 h. Untreated cells were used as controls. At the end of the time, the wells were emptied. The MTT solution prepared at a concentration of 5 mg/mL was diluted 10 times with a culture medium and 100 µL was added to each well. After 4 h of incubation, the wells were emptied and 100 µL of dimethyl sulfoxide (DMSO) was added and left for 10 min [63]. Finally, absorbance was read using ELISA (Biotek Synergy HT) at 570 nm wavelength. Equation (1) was used to compute the percentage of viability relative to untreated controls.

% Viability = [(Abs_sample_ × 100)/Abs_control_] (1)

### 3.5. Statistical Analysis

The Levene test was performed for variance homogeneity. One-way analysis of variance was used among multiple groups. The Dunnett test was applied for multiple comparison tests at *p* < 0.05 level. Quantitative phytochemical analysis experiments were repeated three times and results are expressed as mean ± standard deviation. The non-parametric Spearman’s correlation test was used to assess the correlations between antiproliferative effect and total triterpene, total sterol, total iridoid, and ursolic acid contents of the extracts.

## 4. Conclusions

In summary, the phytochemical content richness and inter-species secondary metabolite variability of *Nepeta* species were comparatively demonstrated in this study. The statistically significant decreases in proliferation of breast cancer cells, especially in the MCF-7 cell line, caused by the *Nepeta* species are striking. Despite a crude herbal extract being generally regarded as having a cytotoxic effect when the IC50 < 20 µg/mL after an incubation period of 48 to 72 h by the US NCI plant screening program, the results obtained from this study are expected to shed light on cancer research that can be carried out with these plants [61]. Considering the statistical analysis, there is a strong correlation between the cytotoxic effects of these plants and their triterpenoid content. However, bioactivity-guided fractionation studies are needed to fully elucidate the compound or compounds responsible for the effect. The cytotoxic effects of plants should be further supported by detailed in vitro biochemical and in vivo studies.

## Figures and Tables

**Table 1:** Total triterpenoid, steroid, iridoid and ursolic acid contents of *Nepeta* extracts.

	Total Iridoid Content (mg Aucubin Equivalents/g Extract)		Total Sterol Content (mg Cholesterol Equivalents/g Extract)		Total Triterpenoid Content (mg Oleanolic acid Equivalents/g Extract)		Ursolic Acid Content (mg/g Extract)	
Species	MeOH	INF	MeOH	INF	MeOH	INF	MeOH	INF
NAT	nd	nd	149.22 ± 2.76	nd	391.91 ± 88.40	18.86 ± 1.70	105.4 ± 0.6	nd
NTX	85.60 ± 4.45	47.12 ± 0.67	115.29 ± 2.29	nd	505.50 ± 96.39	112.00 ± 6.71	22.6 ± 0.6	nd
NCD	nd	nd	89.33 ± 1.26	nd	296.49 ± 12.85	11.59 ± 1.11	32.6 ± 0.4	nd
NCR	33.21 ± 5.05	15.93 ± 0.00	174.16 ± 8.59	nd	603.19 ± 113.00	53.84 ± 3.86	47.3 ± 1.6	nd
NHM	nd	nd	86.67 ± 2.52	nd	537.31 ± 32.61	18.86 ± 3.58	2.7 ± 0.6	nd
NIT	nd	nd	123.05 ± 9.45	nd	503.23 ± 50.50	23.85 ± 4.01	41.2 ± 0.9	nd
NLP	nd	nd	183.97 ± 3.28	nd	448.70 ± 44.98	14.77 ± 1.29	15.5 ± 0.3	nd
NMY	nd	nd	403.09 ± 15.24	nd	464.61 ± 25.50	9.77 ± 1.70	34.8 ± 0.4	nd
NPH	nd	nd	181.92 ± 0.29	nd	928.07 ± 22.26	57.93 ± 1.11	101.2 ± 0.1	nd
NRC	nd	1.29 ± 1.79	180.90 ± 3.00	nd	525.95 ± 11.13	11.13 ± 0.64	165.9 ± 0.6	nd
NSC	5.83 ± 1.68	nd	268.18 ± 6.38	nd	610.01 ± 34.00	9.77 ± 2.32	45.8 ± 2.3	nd
NSS	nd	nd	186.63 ± 6.76	nd	301.03 ± 14.72	0.23 ± 2.32	43.1 ± 0.4	nd
NTC	nd	nd	163.32 ± 6.61	nd	432.80 ± 16.06	7.50 ± 2.23	16.4 ± 0.3	nd
NTR	nd	nd	353.22 ± 12.15	nd	564.57 ± 17.89	nd	28.9 ± 0.1	nd

nd: Not detected. MEOH: Methanolic extract. INF: Infusion.

**Table 2:** Cytotoxic effects of *Nepeta* species on MCF-7 cell line.

MCF-7 % Cell Viability Concentrations (μg/mL)				
Species/Extract	1000	125	3.9	IC_50_
NAT-MeOH	14.30 ± 0.58 ***	63.45 ± 1.52 *	90.08 ± 2.16	186.66 ± 13.32
NAT-INF	23.87 ± 1.25 **	87.68 ± 1.56	102.52 ± 1.70	>250
NTX-MeOH	16.91 ± 0.42 ***	68.26 ± 1.95 *	100.27 ± 0.20	>250
NTX-INF	27.89 ± 1.98 **	95.16 ± 3.21	105.53 ± 1.05	>250
NCD-MeOH	15.88 ± 0.37 ***	77.68 ± 0.37 *	107.37 ± 2.06	>250
NCD-INF	28.77 ± 1.71 **	81.50 ± 2.39	109.37 ± 3.77	>250
NCR-MeOH	14.93 ± 0.23 ***	71.20 ± 0.37 *	102.97 ± 5.02	>250
NCR-INF	54.79 ± 0.51 **	92.02 ± 0.97	110.40 ± 2.97	>250
NSC-MeOH	15.97 ± 0.41 ***	60.25 ± 0.56 **	95.86 ± 1.61	213.14 ± 17.65
NSC-INF	59.12 ± 21.19 **	96.58 ± 3.33	109.42 ± 1.62	>250
NHM-MeOH	18.18 ± 0.44 ***	92.73 ± 0.91	110.19 ± 3.60	>250
NHM-INF	30.27 ± 1.22 **	85.12 ± 1.48	100.94 ± 4.00	>250
NIT-MeOH	23.87 ± 1.25 ***	87.68 ± 1.56	102.52 ± 1.70	>250
NIT-INF	39.85 ± 0.60 **	94.87 ± 0.80	118.93 ± 2.05	>250
NLP-MeOH	15.04 ± 0.24 ***	58.40 ± 2.05 *	101.06 ± 2.37	190.99 ± 13.41
NLP-INF	18.59 ± 1.56 ***	82.72 ± 0.38	116.39 ± 2.48	>250
NMY-MeOH	16.89 ± 0.25 ***	73.44 ± 2.37 *	111.91 ± 1.52	>250
NMY-INF	53.15 ± 1.34 **	93.69 ± 0.73	104.71 ± 2.82	>250
NPH-MeOH	19.16 ± 0.19 ***	40.17 ± 2.57 **	101.57 ± 1.53	79.91 ± 9.47
NPH-INF	15.56 ± 0.82 ***	83.71 ± 1.27	111.65 ± 3.22	>250
NRC-MeOH	16.32 ± 0.30 ***	42.05 ± 0.28 **	91.63 ± 1.38	61.38 ± 1.38
NRC-INF	21.74 ± 0.55 ***	59.72 ± 1.37 **	103.87 ± 0.49	>250
NSS-MeOH	16.55 ± 0.85 ***	63.67 ± 1.57 *	96.77 ± 0.45	>250
NSS-INF	63.45 ± 4.64 *	95.67 ± 1.27	109.57 ± 3.89	>250
NTC-MeOH	16.45 ± 0.06 ***	72.13 ± 1.53 *	102.73 ± 1.97	>250
NTC-INF	41.73 ± 1.20 **	91.88 ± 2.92	123.90 ± 1.43	>250
NTR-MeOH	17.62 ± 0.36 ***	65.55 ± 2.74 *	97.15 ± 2.65	220.66 ± 9.03
NTR-INF	41.01 ± 3.74 **	89.32 ± 3.59	93.33 ± 1.35	>250
	**250**	**31.25**	**0.975**	**IC_50_**
Ursolic acid	9.20 ± 0.38 ***	24.80 ± 0.26 ***	77.84 ± 2.25	29.28 ± 3.48
Cisplatin	9.50 ± 0.09 ***	20.30 ± 0.75 ***	105.07 ± 1.57	69.28 ± 5.55

Data are expressed as mean ± standard error (*n* = 3). Significant differences are indicated as * *p* < 0.05, ** *p* < 0.01, *** *p* < 0.001. MEOH: Methanolic extract. INF: Infusion.

**Table 3:** Cytotoxic effects of *Nepeta* species on MDA-MB-231 cell line.

MDA-MB-231 % Cell Viability Concentrations (μg/mL)				
Species/Extract	1000	125	3.9	IC_50_
NAT-MeOH	5.37 ± 0.05 ***	67.70 ± 2.29 *	117.72 ± 1.32	166.06 ± 7.9
NAT-INF	32.57 ± 2.06 ***	95.93 ± 4.79	105.43 ± 3.69	>250
NTX-MeOH	8.09 ± 0.955 ***	133.47 ± 2.57	131.88 ± 3.03	>250
NTX-INF	29.38 ± 1.16 ***	86.63 ± 1.63 **	113.72 ± 0.89	>250
NCD-MeOH	4.27 ± 0.09 ***	76.63 ± 1.51	96.78 ± 1.91	>250
NCD-INF	30.23 ± 0.13 ***	81.71 ± 1.00	102.41 ± 1.37	>250
NCR-MeOH	4.66 ± 0.09 ***	103.52 ± 1.26	112.11 ± 0. 37	>250
NCR-INF	20.33 ± 0.35 ***	83.86 ± 1.37	105.56 ± 1.72	>250
NSC-MeOH	5.24 ± 0.19 ***	84.13 ± 3.87	107.32 ± 3.53	>250
NSC-INF	59.45 ± 1.55 **	102.42 ± 2.90	100.38 ± 1.13	>250
NHM-MeOH	3.77 ± 0.06 ***	85.68 ± 2.06	105.33 ± 0.76	>250
NHM-INF	38.82 ± 1.92 **	81.93 ± 1.44	105.55 ± 4.23	>250
NIT-MeOH	5.83 ± 0. 14 ***	80.01 ± 0. 48	110.58 ± 0.39	>250
NIT-INF	26.47 ± 0.24 ***	74.60 ± 1.64	110.93 ± 5.28	>250
NLP-MeOH	3.39 ± 0.09 ***	92.59 ± 0.90	102.71 ± 1.38	>250
NLP-INF	40.11 ± 2.24 **	86.61 ± 3.41	122.18 ± 9.19	>250
NMY-MeOH	5.89 ± 0.22 ***	91.22 ± 0.91	116.32 ± 1.47	>250
NMY-INF	38.09 ± 1.94 **	91.80 ± 3.90	109.19 ± 1.23	>250
NPH-MeOH	4.38 ± 0.04 ***	34.74 ± 1.62 **	106.82 ± 2.32	90.80 ± 1.67
NPH-INF	15.47 ± 0.84 ***	86.98 ± 2.91	113.15 ± 0.99	>250
NRC-MeOH	5.03 ± 0.15 ***	19.77 ± 0.91 **	103.86 ± 2.15	94.26 ± 2.85
NRC-INF	37.72 ± 0.37 ***	94.75 ± 2.57	124.94 ± 3.10	>250
NSS-MeOH	28.61 ± 0.59 **	96.69 ± 2.25	97.76 ± 1.47	>250
NSS-INF	72.02 ± 0.90 *	139.57 ± 1.37	142.90 ± 6.28	>250
NTC-MeOH	3.91 ± 0.10 ***	104.82 ± 0.66	109.77 ± 1.32	>250
NTC-INF	23.49 ± 1.11 ***	83.68 ± 4.99	108.02 ± 3.42	>250
NTR-MeOH	6.44 ± 0.10 ***	57.52 ± 1.75 **	112.77 ± 2.09	178.07 ± 9.81
NTR-INF	28.46 ± 0.34 ***	103.96 ± 4.51	125.91 ± 2.33	>250
	**250**	**31.25**	**0.975**	**IC_50_**
Ursolic acid	2.54 ± 0.08 ***	5.52 ± 0.13 ***	83.03 ± 1.78	36.29 ± 2.37
Cisplatin	2.37 ± 0.12 ***	2.37 ± 0.05 ***	30.67 ± 2.33 ***	<0.975

Data are expressed as mean ± standard error (n = 3). Significant differences are indicated as * *p* < 0.05, ** *p* < 0.01, *** *p* < 0.001. MEOH: Methanolic extract. INF: Infusion.

## Data Availability

The data presented in this study are available in the article and no supplementary material has been added.
